# Cancer, a relational disease *exploring the needs of relatives to cancer patients*

**DOI:** 10.1080/17482631.2019.1622354

**Published:** 2019-05-23

**Authors:** Ulrika Sandén, Fredrik Nilsson, Hans Thulesius, Maria Hägglund, Lars Harrysson

**Affiliations:** a Department of Design sciences, Lund University, Lund, Sweden; b Research and Development Kronoberg and Linnaeus University, Växjö, Sweden; c Department of Women’s and Children’s Health, Uppsala University and Uppsala University Hospital, Uppsala, Sweden; d School of Social Work, Lund University, Helsingborg, Sweden

**Keywords:** Health, family members, carers, cancer care, grounded theory, waiting, safety, momentary contentment, salutogenesis, informal caregiver

## Abstract

**Purpose**: In this qualitative interview study we investigated the experiences of family members to cancer patients. Our objective was to explore and to differentiate their needs from the needs of cancer patients.

**Methods**: Five focus groups and six individual narrative interviews with 17 family members to cancer patients in Sweden were conducted and compared with 19 cancer patient interviews. Our analysis was inspired by classic grounded theory.

**Results**: Family members to cancer patients expressed own morbidity connected to high stress levels and difficulties in recognizing own stress due to ongoing comparisons with the cancer patient. Family members were trapped in a momentary terror-like situation where they became their sick relative’s safety net. A percieved inability to improve their loved one’s well being contributed to a feeling of guilt. The longing for it all to end was encumbered with shame since the end included possible death.

**Conclusions**: By recognizing cancer as a disease striking both body and relationships, family members are given precedence over their own struggles, differentiated from the patient’s experiences. We define differences in needs between cancer patients and family members. Family members to cancer patients may be supported in developing balancing strategies towards less stress, increased safety and moments of contentment.

## Introduction

Since more people survive cancer and more live longer with a chronic disease (Siegel, Miller, & Jemal, 2019) there is also an increase in family members living with cancer. Spouse caregivers show morbidity connected to high levels of stress, anxiety, potential burnout, depressive symptoms, marital distress, poor health, and unmet needs (Braun, Mikulincer, Rydall, Walsh, & Rodin, 2007; Goren, Gilloteau, Lees, & DiBonaventura, 2014; Lehto, Aromaa, & Tammela, 2018; Li & Loke, 2013; Sandén, 2017; Sjövall, 2011). Cancer is an illness that often requires family members to engage. Cancer is on the increase and in Sweden we struggle with providing psychosocial support to both patients and family members. Migration and possible language barriers adds to the challenge (Sethi, Williams, Zhu, Shen, & Ireson, 2017). In order to be able to provide support to affected families, there is a general need to increase and adapt family support to this emerging situation of a slowly less fatal disease.

When cancer strikes a patient it promptly becomes a health problem to fix, but when family members are struck through their relationship with the patient it eventually becomes a problem for society as increased morbidity. However, family members’ needs start long before it becomes a societal problem. Jussila (2008) and Andreassen, Randers, Nyhlin, and Mattiasson (2007) argues the inclusion of family in caring for the patient. At the same time as we agree, we argue the importance of allowing the family members to have their own relationship to the disease with varying needs and different experiences of the disease. We see a need for easy access to userfriendly self-help tools to increase safety, moments of joy and relaxation in a stressful situation.

In this study we firstly wanted to explore the experiences of family members to cancer patients. Secondly, our aim was to find ways to handle the presumed distress caused by a relationship affected by a severe disease such as cancer. Many health theories, such as Salutogenic theory (Antonovsky, 1996), Self-efficacy theory (Bandura, 1997) and Momentary contentment theory (Sandén, 2014, 2017), align in their use of coping strategies but differ in their view on control, meaningfulness and individualism. The former two are commonly referred to as implemented in Swedish health care while the latter is newer and not yet clinically tested. The salutogenic theory argue a sense of coherence, found through meaningfulness, comprehensibility and manageability. Self-efficacy has its focus on the cognitive process within the individual and his/her view on one’s personal ability to take control and to cope in a situation. The momentary contentment theory is based on an acceptance of life as being hard and it uses three safety balancing mechanisms: Doing safety, Destiny readiness and Middle consciousness. The *meaning of hardships* is central in both salutogenic and momentary contentment theories but in different ways. Where salutogenic theory focuses on finding a meaning with what happens in life, momentary contentment theory focuses on enjoying life and an acceptance of that there often is no meaning. *Comprehensibility* is elaborated in both the self-efficacy and the salutogenic theory. In the salutogenic theory you get a sense of prediction and trust that things happen in an orderly fashion, whereas the self-efficacy theory focuses on the individual’s belief in her own ability. *Manageability* is included in all three theories. The salutogenic as well as the self-efficacy theory have the individual as a focal point, whereas momentary contentment theory focuses on the group level. Our emerging objective was to illuminate ways of supporting the development of strategies to enhance psychosocial wellbeing among family members to cancer patients.

Our research questions were: What are the needs of family members to cancer patients? How do salutogenic, self efficacy and momentary contentment health theories express ways of meeting those needs? In this study the objective was to answer these questions and to suggest possible solutions to the main concern of the participants.

## Method

### Interviews and empirical analysis

This qualitative study builds on narrative interviews with 17 family members to cancer patients. All informants and their cancer sick relatives were above 18 years of age. They were interviewed in five focus groups complemented with six individual interviews. The interviews lasted between two and three hours each. For 15 interview participants their cancer sick loved one was still alive. We informed about the study through advertisments in social media and at primary care facilities. We asked to interview people over 18 years of age experiencing cancer as a loved one. Both parents, spouses and children to cancer sick patients responded and were interviewed. We regarded it as a strength to allow diversity in relationships. The patterns found are thus usable on a variety of family members. Further research may pin point specific needs to specific family members. New data was collected and analyzed until further data did not provide any new information and saturation was reached. All interviewees wanting to participate were accepted until enough informants to finalize the study was reached.

Unstructured narrative interviews were used as a way to let the informants decide what was important to share. It is a mode to gather data both based on what participants say, how they say it and what they choose to talk and not talk about. The method is useful when focus is on experiences revealed only when informants tell a story in their own way (Gillham, 2008). Carefully detailed field notes were written during interviews, but in accordance with classic grounded theory (Glaser, 1998) no audio recordings followed by verbatim transcriptions were done.

Our analysis was conducted with the methodological features of classic grounded theory, yet we do not claim to have reached the asymptote of a fully integrated grounded theory (Glaser, 1978). The analysis include data collection, coding, comparison and categorization. Classic grounded theory aims at explaining and conceptualizing what is going on in a substantive area. Each interview was field noted, then coded and then compared to previous interviews. Theoretical memos, a crucial part of classic grounded theory, were written after and between interviews as well as in the analysing phases, then coded and categorized and eventually sorted. Memos are a central part of the data material in accordance with classic grounded theory (Glaser, 1998). Doing a grounded theory study is a circular process of constantly comparing, coding and analyzing new data until it does not provide any new information (Glaser, 1998). The concepts were gradually developed to explain the informants’ attitudes toward life. After the core category of navigating was finalised, memos and notes were written without discrimination, but interpretation and analysis was done selectively guided by the core category. When no new information was reached through data collection all memos were compared and sorted to find relationships between categories and concepts. Data from family members was further compared to data from a previous study (Sandén, Harrysson, Thulesius, & Nilsson, 2017) where 19 cancer patients were interviewed. What has emerged from the write up of the sorted memos so far is not a saturated grounded theory but a conceptual description called “Navigating cancer as a loved one”. We have previously described a theoretical fit between the patient study “Navigating a new life situation” and Momentary contentment theory (Sandén et al., 2017). In this article we do the same with relatives to cancer patients. Clinical studies need to be done to explore if and how the momentary contentment theory need to be revised in order for a grounded theory of cancer navigation to emerge.

The Regional Ethical Review Board at Lund University approved the study (Reg nr 2016:219).

### Fit, relevance, workability, modifiability

The results in a grounded theory study are not reports of facts but rather probability statements about the relationship between concepts or an integrated set of conceptual hypotheses developed from empirical data. Grounded theory is thus judged by fit, relevance, workability, and modifiability (Glaser, 1998, p. 18). Fit and relevance were achieved through the continuous comparative analytical work. By focusing on what the informants chose as important topics such as information, communication and the overall pressure we thereby allowed the main categories to emerge through conceptualization. The informants were offered to read and comment on the analysis to ensure both fit and relevance. The workability is seen in how the core category of navigating explains what participants are doing to resolve their main concern. The workability of the core category is also seen in how the different categories all involve navigating in some way. The study has not lead to a new theory and has thus no workability in its own. Modifiability of the study was performed by connecting it to the cancer patient study and to health theories.

## Results

Family members to cancer patients described a situation where guilt and problems in healthcare put demands on them to be alert at all times. Expressions like *“I didn’t dare to get sick, who would take care of everything?”* and *“I became the safety-net”* show how the relatives put pressure on themselves to be strong and to put their own needs secondarily to the patient’s. Cancer patients, when put in groups with the same diagnosis, tended to form a hierarchial order of seriousness of the disease between themselves (Sandén, 2016). This was less common among family members in our study, but when it happened, the hierarchy also followed the seriousness of the cancer, not the actual situation for them as relatives. Stress related morbidity became a part of life for family members in our study, and we wanted to provide concrete suggestions for strategies to enhance wellbeing.

### Momentary terror and time juggling

Our interviews showed how family members rarely received help based on their needs, or help to express their needs. Instead their lives were tinged by obligations. They found themselves in a fragmented healthcare process expected to manage like a project leader without training or systematic support. Bandura’s (1997) self-efﬁcacy theory suggests that lack of perceived control is underpinning most forms of anxiety. Many interviewed family members expressed powerlessness towards the disease and its potentially deadly outcome. In addition family members lacked a language to describe their role in life. They used “parent” “helper” “project leader” “nurse” in attempts to capture their situation. Fivush and Merrill (2014) argue the role of language as in naming and conceptualizing experiences and feelings and thus the abstract can be made tangible. The interviewed family members struggled with reflecting upon their own situation and many experienced a terror-like momentary situation. In our earlier interviews with cancer patients many expressed an ability to separate healthy parts of their lives from the sick, and how they actually enjoyed these episodes (Sandén et al., 2017). Family members did not seem to have the same ability to juggle time, but were rather in a mood of almost constant alert. To constantly face illness and the need to both guard and manoeuvre the healthcare process takes over the present moment. Many described how they never dared to disconnect from the disease: “*One must always be on guard for possible mistakes*”.

When asked about needs, they kept returning to the patient’s experiences. They were longing for it all to end, but the longing was hammered with guilt due to the unsure future of the patient. Many testified that the cancer sick person would not have survived without their effort and they were scared to let go of the constant control. Several family members described relief when resourceful palliative care arrived. Suddenly the healthcare system worked and they could somewhat relax a bit. It became evident that family members often lacked recognition for their work; linguistically, judicially and economically. Instead high morbidity and sick leave (Sjövall et al., 2009) show how society use this resource in an unsustainable way.

### Doing safety

Patients “did safety” with other patients through organizations, websites, and in relationships with family and healthcare staff. Family members showed no such sense of “doing safety”. Rather they expressed a need to actually be the safety net—*“I was too afraid to get sick, who would then handle everything”*. Lack of safety feelings and communion among family members made them struggle. Antonovsky (1996) stresses the need to feel a sense of coherence with what happens, something which is very difficult when the family members do not see their own needs. Knowing our place in context over time affects our inner sense of continuity and in this lies a feeling of security (Rämgård, 2006). Living with a person sick from cancer makes it difficult to feel safe in a relational continuity. Family members seemed to have no safety net and difficulties in separating themselves from the fear of cancer. They expressed needs on a general level, often arguing *“(the) health care needs to see us, we have needs*” but when asked about personal needs the most common answer was “*I don’t need help personally*”. It was as if they did not see their own needs other than through their sick loved one. When healthcare resources were increased, family members experienced clusters of safety enhancing moments, which made other existing needs legitimate.

### Delegitimation

Family members expressed with anger and pain how the patient sometimes got delegitimised. However, they also added to their own delegitimization by not recognizing their personal situation as painful or by adding guilt when in pain without being the one with a tumor. Ware (1992) discusses *delegitimation* experiences as when disease or pain is denied. She mentions two forms; one where people suppress experiences of illness with words like “we are all tired”, and another where physicians define the experienced illness as psychosomatic. Both types means a questioning of a person’s ways of thinking. However, according to Ware (1992), the second is more damaging to the patient since it includes a psychiatric illness, containing strong stigma . Several informants said that “family members need support and help”, but they also answered the question “what are your needs?” with “*I don’t have any needs, but other family members do*”. This show how family members deny themselves of help. According to self-efficacy theory people’s ability to cope with stress is linked to their belief in themselves (Bandura, 1997). Healthcare organizations need to help family members see themselves as important. Evergeti (2011) mentions how people reconstruct their images of the stigmatized self, how complex interactions reinforce homogeneity in one group and, in interactions with members of other groups, maintain differences. She shows how a reaction to discriminatory and socially excluding conditions is a significant personal identity marker as opposed to a dominant society. If being the safety net becomes an identity marker it may explain why family members show difficulties expressing their needs separated from those of their cancer sick loved one despite knowing a need exists on a generalized level.

### Time horizons

“*Time is nature’s way to keep everything from happening all at once*” (Wheeler, 1990, p. 10; Cummings, 1922). The social perception of time distinguishes one culture from another. Local appreciation of time within a community is often unstated, thus difficult for outsiders to perceive (Levine, 1996). Time is an issue for chronically ill people, both in regard to the disease, and to the various actions that have to be directed in consequence of the disease (Gunnarsson, 2016). Both patients and family members struggled with waiting. Waiting for answers, waiting for x-ray, waiting for treatments and waiting in waiting rooms. Both Gunnarsson (2016) and Auyero (2011) illustrated a process where patients and social recipients learn to be patient. Auyero (2011) notes that ”*collective time senses are deeply intertwined with the workings of (and resistance to) social domination*” (p. 7). He lifts time as the locus of conflict, but also, and as important, of acquiescence.

If life itself is one cluster of moments, it is generally seen as shorter by patients than by family members. One explanation is that when looking at a possible death there is no imaginary life beyond that point for the patient. However to the family members there still is a life after a possible death, thus the horizon was closer to most patients. Patients and family members showed difficulties in recognizing each other’s different time perceptions. Among family members it was apparent that the present moment was characterized by anxiety and guilt: “I was ashamed, I wasn’t allowed to feel bad”. Patients, on the other hand, had a shorter time horizon not knowing whether they would survive or not, and they experienced more moments of joy. Macduff (2006) discusses the impact of different perceptions of time on the priorities given to past, present, and future orientations. Two key dimensions in negotiating across cultural boarders are lifted: i/differing perceptions and values of time, and ii/management of time. In a cancer context, with life and death in proximity, the perception of time changes in different ways for patients and family members. Macduff’s (2006) arguments illustrate possible causes for misunderstandings in families.

Patients seem better equipped to juggle time and to disconnect from the disease and enjoy moments, thus creating new small clusters of moments. Nordenfelt (2005) describes phenomenologically how chronically ill people create parallel worlds such as that of sickness, a medical world and an everyday world. Many interviewed patients who were done with treatments described how they separated the fear of illness and death they felt during the waiting time between an examination, such as an imaging procedure, and receiving the results of the examination, from the rest of their life. They capsulated fear as a way to put the fear of dying in a cluster of moments and thus separating the illness from the healthy part of oneself. A framed space of fear is created and contributes to safety feelings outside that frame. Family members showed no such strategies, rather they uncontrollably accepted the illness as one big space of fear, longing for it all to end. This, in some cases, means that the death of their family member exaggerates feelings of guilt. “I almost look forward to palliative care”.

### Finding a balance?

Malterud and Hollnagel (2004) refers to Antonovsky and patients’ sense of coherence in finding coping strategies for health when faced with illness. Boscherini (2017) explains Antonovsky´s salutogenesis in terms of keeping it together in the face of adversity, manifested in the availability of resources, a supportive social network, order and familiarity and in an inspiring realization that there are important phenomena in life and nature. The philosopher Bruce N Waller (2002) argue the need for both an internal locus of control and a sense of competent self-efficacy when discussing the psychological structure of patient autonomy. Waller (p. 257) states “Those with an internal locus of control believe that their life’s course is basically up to them”. The interviewed relatives in our study described how their life had almost been hijacked by the disease. They also talked about being placed in a situation without proper training or knowledge, thus lacking self-efficacy:

*“You get into a double-mindedness. On the one hand, you should be a fixer, the one who can do everything, be both a nurse, an occupational therapist and a curator at the same time. On the other hand, there is no access to contact facilities with healthcare, one has to wait for other people’s decisions and hope to be taken seriously*.”

The fact that relatives don’t have the disease in their body makes it more difficult for them to balance their fear. Wallhagen and Brod (1997) showed how perceived control over symptoms had greater influence over well-being than beliefs concerning ability to control the disease itself (Wallhagen & Brod, 1997). According to Waller (2002), if one loses control in one area it may be balanced by increasing control somewhere else. In a study on mental health, researchers tried to understand the Samoan individual. In interviews they called themselves “a relational self” and mental health for “relational harmony and balance “. Another approach found in the study was the perception of well-being and how the well-being of the individual was protected by means of relational agreements with others (Tamasese, Peteru, Waldegrave, & Bush, 2005). This connects to the momentary contentment theory (Sandén, 2014) where safety is built on balancing mechanisms where individuals act within a community and adversity is faced together. To balance adversity in different ways seem to be a part of coping mechanisms in different theories. Looking at our interviews with relatives and cancer patients, balance was hard for the relatives to find.

## Discussion

We have previously interviewed cancer patients and for this study family members to cancer patients. For this article we have compared the results from both interview studies, but with a family member perspective. In many interviews, focus groups and one-on-one, family members to cancer patients, described a lonely situation where they were stuck in a terror-like situation with few breaks. Several participants expressed how they before meeting other relatives, believed they were becoming mentally ill due to their strong feelings of fear, anxiety and stress. Further, literature show morbidity among relatives and we argue a need to widen the concept of cancer where family members are affected relationally aside from the patient.

### Cancer as a relational disease

Both cancer patients and family members saw cancer as a disease striking patients first and family members second, presenting a power imbalance giving patients interpretative precedence. Family members expressed more loneliness than patients did. Some felt guilt when having fun. They were *doing safety* by helping, but this was described as a lonesome effort where they never seemed to be a part of a shared collective *doing safety*. Guilt came from the notion of cancer not affecting family members directly, but through the patient. At the same time this created guilt for patients, being the carrier of the pain, i.e., the cancer. If we rather view cancer as striking everyone it meets, but differently, then family members are affected in a relational way, whereas patients are affected both bodily and relationally. If we view cancer as both a relational and somatic disease it does not give precedence to feelings of neither patients nor family members. Family members often expressed a general need for help, but seemed unable to define any needs. They had difficulties talking about their own lives with cancer without allowing the patient in between themselves and the disease.

Healthcare devote few resources directly to family members, instead they use resources due to morbidity connected to the high stress of being an informal care-giver (Goren et al., 2014). A more inclusive healthcare, asks for a changed approach to both patient processes and in regard to a broader participation of the patient’s social network. Intercultural studies add language barriers as another burden to the caregiving role (Sethi et al., 2017) which also has to be considered. Goren et al (2014) states “*the need for enhancing our understanding of the caregiving experience and developing supportive and personalized multicomponent interventions for caregivers, given their pivotal role in providing support for patients*” (p. 1637). It is thus a wicked problem as defined by Rittel and Webber (1973) where both mindset, behaviour patterns and multiple societal factors are involved, and a solution would be complex. Thakur, Hsu, and Fontenot (2012) stresses the importance of understanding the challenges faced by healthcare organizations, e.g., multiple medical records of patients and incorrect doses of drugs and wrong medication. Our study show how the consequences of such challenges, when healthcare fails, are placed on the family members to handle, making them unable to relax from the stress of being the safety net.

### From guilt to pride

Momentary contentment theory shows language use as a coping strategy in reformulating problems into solutions, and, alongside, place an initial problem in a temporal state of mind—*Middle consciousness* (Sandén, Thulesius, & Harrysson, 2015) where one may use symbolic opposites to find balance. Patients already juggle between feeling sick and healthy in using symbols of health in their daily life (Sand, Olsson, & Strang, 2009; Sandén, 2017). As patients oscillate between feeling sick and healthy, family members have other opposites. Many family members expressed being alone with thoughts and feelings. They described guilt from having fun when their loved one was sick, a cognitive process where they added more guilt on themselves and quite the opposite to *Destiny readiness*, which means accepting life as it is. In some cases that means accepting death. Jonasson et al. (2011) studied men’s feelings of guilt after their wife’s death in cancer and concluded the importance of having end-of-life discussions within the last 3 months before her death. An opposite to guilt could be pride. Thinking “our relationship has cancer” can give legitimization to guilt and self-pity, as we are in this together—but also to pride and joy, for the navigating work that is being done. Momentary contentment strategies may be used to find a balance between being a caretaker and a project leader, and finding a workable life where there is space for just being in the relationship in spite of the disease.

### The creation of cluster of moments

Mishra, Brakey, Kano, Nedjat-Haiem, and Sussmanbc (2018) asked young adult cancer patients and their primary informal caregiver about factors that made a “good day”. The cancer patients referenced normalizing activities such as doing chores, leaving the house, and seeing friends and family. Whereas the caregivers did not let go of the disease but rather considered good days in relation to their loved ones’ well-being, such as when they are “feeling good” and “not sick”. Sand et al. (2009) showed how patients in palliative care developed useful strategies to hold off death. They described it as a cognitive and emotional pendulum that swing between extremes, where patients use the tools that suit their own concepts. Thulesius, Håkansson, and Petersson (2003) theory of equilibrium of hope shows how people create instinctive compensatory strategies to increase existential hope, such as a denial of life-shortening information or by increasing momentary enjoyments of life. We saw no such strategies among family members. In the Momentary contentment theory (Sandén, 2017), the concept *momentary* is defined as a subjective formation of clusters of moments. By dividing time into clusters of moments the burden of stressful experiences may lessen. Through separating activity and waiting into different clusters of moments one may break the waiting anxiety during part of the waiting periods. If done in communion with others a joint collective safety-net may evolve. Letting both patients and family members know when an answer is expected can create predictability. This is well in line with Lasane and O’Donnell (2005) argument that people in the Western world need to establish and relate themselves in a temporal perspective, a need to find coherence, regularity and predictability. Momentary contentment theory shows how time can be viewed from various references on a collective basis (Sandén, 2014).

### From loneliness to altruistic communion

Altruism is central to life, past and present, in an interdependent relationship of those helping and those being helped. In groups, altruism means shared pride based on partially unconscious processes wherein it is hard to see our own role. Momentary contentment theory illustrates strategies of helpfulness in illuminating altruistic patterns among patients and family members. It shows the need to balance the helping in shared helpfulness, i.e., helping in the helping. Helpfulness as action and altruism as phenomenon are thus vital. Safety is created in the trust of not having to face difficulties alone (Sandén, 2014). Post (2005) describes how altruism results in positive social inclusion, in distraction from personal problems and self-centred anxiety, in increased wellbeing combined with experiences of meaning and purpose and in a more active lifestyle. A strong link is found between altruism and wellbeing, happiness, health and longevity—as long as helping others does not overwhelm a person. The help family members give, enhancing others’ wellbeing, may be argued as altruistic. However, it is clear that many family members are overwhelmed and show stress and fatigue, rather than wellbeing (Sandén, 2017). Leonidou and Giannousib (2018) describe family caregivers’ experienced responsibility for bringing together medical information received from different sources, organizing notes for the patient and transferring information from one doctor to another to receive the best possible healthcare. Self-imposed demands to create stability, order and safety for the sick relative also seem to stem from a touch of guilt (Sandén, 2017; Sjövall, 2011). Patients on the other hand yearn for altruistic actions, but lack strength to follow them through. Many cancer patients find ways to use activity and helpfulness as tools for feeling better. However, they express difficulties in balancing receiving and giving help (Sandén, 2017). Both patients and family members need support in balancing their engagement.

### Finding hope within

Within the concept of hope patients express uncertainties, misunderstandings and inconsistencies (Sandén et al., 2017). They convey a hope that “lives within”, an opening for *destiny readiness*, but many also feel pushed by relatives, friends and healthcare staff towards being positive and cognitively hopeful. The latter usually includes moving focus towards a change and thus to the future, a future which to a cancer sick person also means fear of death. This is adjacent to Benzein, Norberg, and Saveman (2001) distinction between “living with hope” and “to hope for something”. The former relating to what is present, the latter to a changeable future. Our interviews show the importance of offering hope, and to do it in compliance with life as is (Sandén, 2006, 2017).

Family members are living and acting in the moment, but since the moment often is filled with suffering they evidently wish for a future when the cancer is gone. In line with both Benzein et al. (2001) and momentary contentment theory we think that a cognitive factor of the future puts family members more in a “hoping for” mode, whereas patients are more in a “living with hope” state of mind (Sandén, 2017). To internalize the family members’ “hoping for” mode into “living with hope” their momentary situation should include more of being themselves rather than being the safety net and project leader. In order to not create morbidity, it is important to find balance in the caretaker role.

### Implications and suggestions for practice

*We argue that healthcare stakeholders could do the following to suggest possible solutions to the main concern of the participants*:

- help family members get in contact with other family members of cancer patients.
- recognize that family members have feelings separate from the person carrying the disease.
- recognize family members as affected by the disease and give them support helping them to go from guilt to pride and support them in taking care of their own needs. Teach them the difference between hoping for and carrying a hope within.
- Most importantly, we suggest that healthcare staff should recognize that family members’ need to have a life of their own, and, importantly, they cannot be the continuous safety net for healthcare mistakes, errors and mishaps.

### Limitations and future research

Limitations of our study sit mainly in the number of participants interviewed. Also, the regional generalizability is limited since all participants came from three different regions in Sweden. In future research a survey could be designed from the results of this interview study to explore its generalizability. Each need may also be further explored, contextualized and given new solutions.

## Conclusion

Cancer affects patients and family members in different ways. The patients are physically affected, leading to relational difficulties, while the relatives are relationally affected and develop different types of morbidity.

Our interviews showed a situation where patients were seen as the primary interpreter of the disease and its impact on life. However, by recognizing cancer as a relational disease alongside with the tumor, every person affected by cancer may be given precedence over his/her own experiences and needs. Then by recognizing the differences in experiences and needs, existing and potential misunderstandings between patients and family members can be dealt with.

Skills found in different health theories may be applied in the search for better health. For example: sharing various fears of dying or being left alone, of different time horizons, and of the concept of hope versus hoping for.

The literature shows high morbidity for people living close to a cancer patient. Patients and family members have disparate needs, but they share an overall longing for a less fragmented healthcare.

Both cancer patients and family members show difficulties in expressing the specific needs that comes from being in a relationship affected by cancer. We have been able to illuminate a few specific needs.

- Family members have needs of their own, but lack insight and concepts to describe them.
- Morbidity among family members to cancer patients may be lessened by applying health promoting strategies.
- Cancer is not just a bodily disease, but a relational disease as well.

We finally argue the need for a recognition of cancer as a relational disease creating morbidity in family members. To meet these needs momentary contentment theory, as a theory built on communion and helpfulness, may be useful. In a collective setting, family members can help each other to create time and space for safety, a break from demands and a relief of pains.
